# Osteological differences in the humerus of loggerhead and green turtles

**DOI:** 10.7717/peerj.20958

**Published:** 2026-04-20

**Authors:** Il-Kook Park, Suhwan Yoon, Hae Rim Lee, Young Jun Kim, Hee-Jin Noh, Sang Hee Hong, Min-Seop Kim, Dongwoo Yang, Il-Hun Kim

**Affiliations:** 1 Marine Biodiversity Division, National Marine Biodiversity Institute of Korea, Seocheon, Republic of South Korea; 2 School of Biological Science and Biotechnology, Chonnam National University, Gwangju, Republic of South Korea; 3 Division of Zoological Research and Management, National Institute of Ecology, Seocheon, Republic of South Korea; 4 Ecological Risk Research Department, Korea Institute of Ocean Science and Technology, Geoje, Republic of South Korea; 5 Department of Biomaterial Research, National Marine Biodiversity Institute of Korea, Seocheon, Republic of South Korea

**Keywords:** Flipper, Humerus, Interspecific difference, Migration, Sea turtle, Size estimation

## Abstract

Limbs are the principal locomotory structures in vertebrate body, closely related to the habitat use and locomotion strategies of the species. Sea turtles rely almost entirely on their fore flippers for aquatic locomotion. The humerus is the most crucial bone structure connecting the scapulocoracoid and flipper, and studies thereof are used to understand turtles’ locomotion mechanisms and swimming strategies. To define the morphological traits of the humerus of loggerhead (*Caretta caretta*) and green (*Chelonia mydas*) turtles and analyze interspecific differences, sea turtle carcasses were sampled from strandings and bycatch in Korean waters within their northern range limit. The length and width of the carapace, plastron, and 15 parts of the left humerus from the carcasses were measured and analyzed. The humerus length and width were positively linearly correlated with the straight carapace length of both sea turtle species, indicating that the carapace size of sea turtles can be reasonably estimated based on the size of the humerus. Additionally, significant interspecific differences were identified in 12 humeral segments, with loggerhead turtles having a humerus that was longer overall but thinner toward the shoulder than green turtles. These differences may be related to their migration patterns, as loggerhead turtles, which migrate long distances, have elongated humeri that are wider toward the tip, which may reflect their efficient use of ocean currents for swimming instead of relying on flapping. In contrast, the green turtle, which is a more vigorous swimmer, has a short but thick humerus that can withstand the stress on the shoulder caused by flapping. These findings demonstrate that bone structure and function differ among even closely related species, depending on their habitat and the environmental exploitation strategies employed.

## Introduction

Morphological traits are the result of evolution optimized for an organism’s environment and ecosystem, and reflect their functional performance (Aerts et al., 2000). Animal limbs are the principal structures for locomotion and support of body mass (Alexander, 1993; Christiansen, 2002). However, understanding the functional structure of limbs, depending solely on investigating the appearance of the species, has limitations. Various methods, including osteological, anatomical, and mechanical approaches, have been developed to gain a deeper understanding of the functional mechanisms of an organism’s limbs. In particular, osteological approaches can improve our understanding of structural and mechanical functions (Marcus et al., 2009; Dickson & Pierce, 2019).

The humerus is a long bone that extends from the scapulocoracoid to the elbow. It serves as a connection point between the distal part of the limbs and shoulders by attaching the humeral head to the scapulocoracoid, playing a crucial role in locomotion (Depecker et al., 2006). Hence, the humerus is frequently used to study the functional characteristics of turtle limbs (Nakajima, Hirayama & Endo, 2014; Dickson & Pierce, 2019; Abell et al., 2023). Turtles (Order Testudinata) are divided into four groups based on their primary habitat types and lifestyles: terrestrial, semi-aquatic, aquatic, and marine. Each group has distinct types of limbs (Nakajima, Hirayama & Endo, 2014; Dickson & Pierce, 2019). As the degree of land use increases (*e.g*., in tortoises), the limbs are more suited to supporting the body’s weight and walking, whereas they become more suited for swimming as the degree of water use increases (Abdala, Manzano & Herrel, 2008; Dickson & Pierce, 2019).

Sea turtles spend their entire lifetime in the ocean except for the period immediately after hatching and when they come ashore to lay eggs. Thus, sea turtle forelimbs are primarily adapted for locomotion rather than supporting their bodies (Nakajima, Hirayama & Endo, 2014; Dickson & Pierce, 2019). Their limbs are broadly modified, resembling oars, and are called flippers. With their broad and solid carapace, sea turtles rely entirely on flippers for locomotion compared to other marine vertebrates that swim primarily by pelvic, pectoral, and caudal oscillations because of their thin and flexible bodies (Sfakiotakis, Lane & Davies, 1999; Davis, 2019). These sea turtle flipper modifications are optimized for energy use, enabling flippers to generate propulsion with minimal flapping and ride sea currents (Kinoshita et al., 2021; van der Geest et al., 2022). Consequently, sea turtles have relatively straight and dorsoventrally flattened humeri compared with other turtles on land (Dickson & Pierce, 2019; Hermanson et al., 2024).

Considering these characteristics, the humerus has been analyzed primarily to understand the locomotion and ecology of sea turtles in the ocean (Romer, 1956; Nishizawa, Asahara & Kamezaki, 2013; Nakajima, Hirayama & Endo, 2014; Abell et al., 2023). Several studies have been conducted on the sea turtle humerus, including shape structure (Abell et al., 2023), bone density (Nakajima, Hirayama & Endo, 2014), phylogenetics (Dickson & Pierce, 2019), and age estimation (Avens & Goshe, 2007). Comparisons between terrestrial and aquatic turtles have also been undertaken. However, comparisons between sea turtle species are still lacking. The interspecific dimorphism of specific osteological structures could explain the functional performance and adaptation to the environment of the relevant part in detail (Ponssa, Brusquetti & Souza, 2011). Nishizawa, Asahara & Kamezaki (2013) compared the humeri of loggerhead (*Caretta caretta*) and green (*Chelonia mydas*) turtles, but used only the total length and two measurements of the humerus diameter. Therefore, analyzing the morphology and comparing several parts of the humeri of the two sea turtle species can enhance our understanding of their adaptations and functions.

This study aimed to provide diverse humeral morphometric traits of loggerhead and green turtles, the dominant sea turtle species in Korean waters (Kim et al., 2017, 2021), and compare interspecific differences in humerus shape. Furthermore, we inferred the ecological factors related to the interspecific differences in the humerus. Additionally, the correlation between humerus and carapace size was analyzed to estimate the size of damaged or decayed sea turtles based on the humerus.

## Materials and Methods

### Sample collection

**Figure 1:**
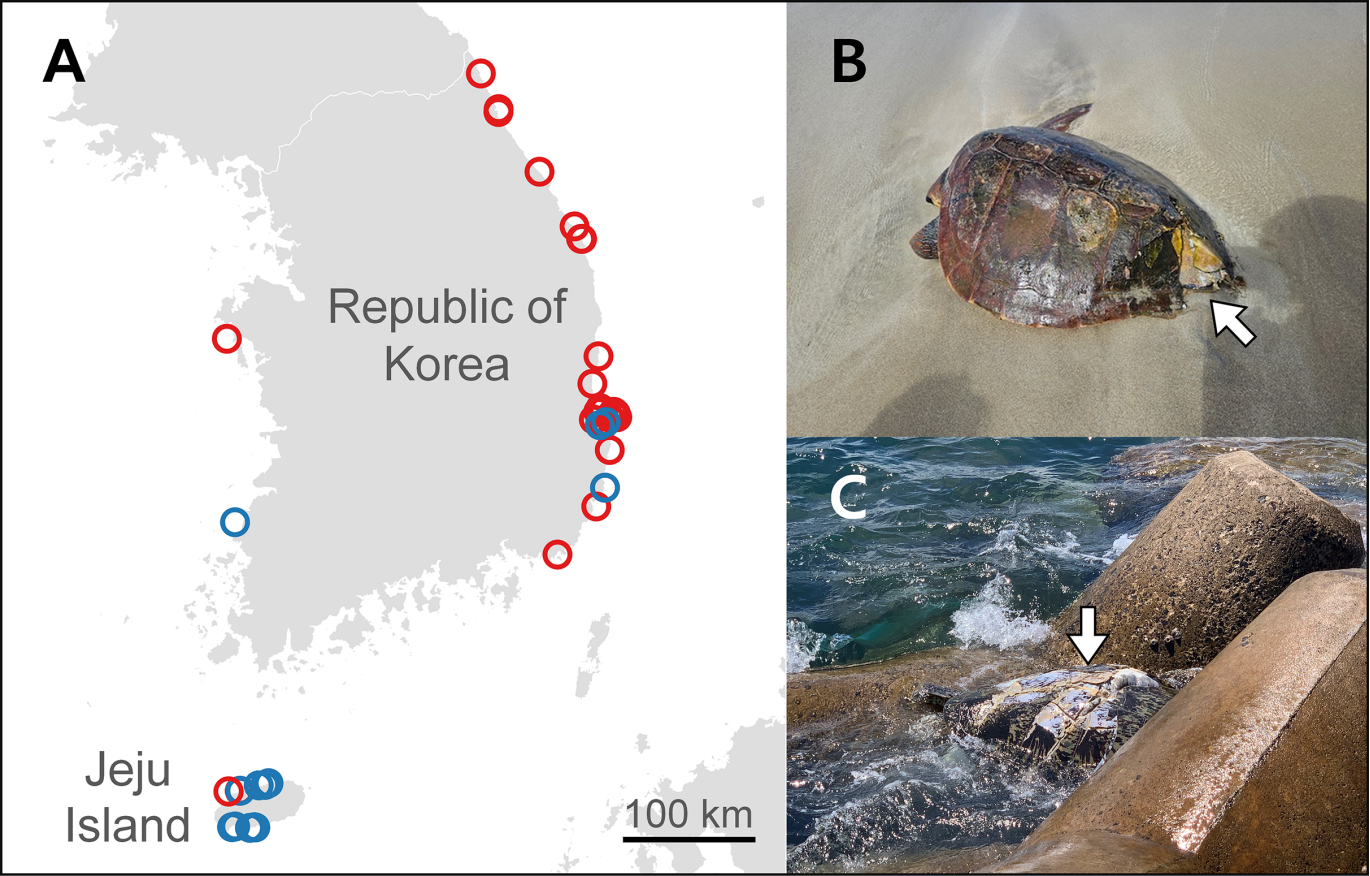
Sampling sites and damaged sea turtle carcasses. (A) Occurrences of loggerhead (red circles) and green (blue circles) turtles. (B) A loggerhead turtle with a damaged back carapace stranded on the shore of the Korean Peninsula, and (C) a green turtle with a broken carapace found floating.

Between 2014 and 2024, we collected 42 carcasses of sea turtles caught as bycatch or that were stranded (28 loggerhead and 14 green turtles) on the coastlines of the Korean Peninsula and Jeju Island (Fig. 1). Sea turtle occurrences were reported by members of the local government, maritime police, institutions specializing in rescuing and treating marine animals, and citizens. We transferred the samples to the National Marine Biodiversity Institute of Korea in Seocheon, Republic of Korea, and stored them in a freezer at –20 °C to prevent deterioration. We measured the straight carapace length (SCL), straight carapace width (SCW), curved plastron length (CPL), and curved plastron width (CPW) of each turtle in units of 0.1 cm. We did not analyze the carcass weight because the time elapsed between the turtle’s death and measurement could have caused a high error rate. Additionally, we determined the sex of the turtles based on their gonads through autopsy or using physical characteristics such as tail length and front claws, which were more pronounced in males when the SCL exceeded 60 cm (Wibbels et al., 1991; Ishihara & Kamezaki, 2011). Although the criteria for sea turtle maturity vary across studies, we classified individuals with an SCL of 60 cm or less as juveniles because their gonads were not fully developed.

### Measurement of the humerus

**Figure 2:**
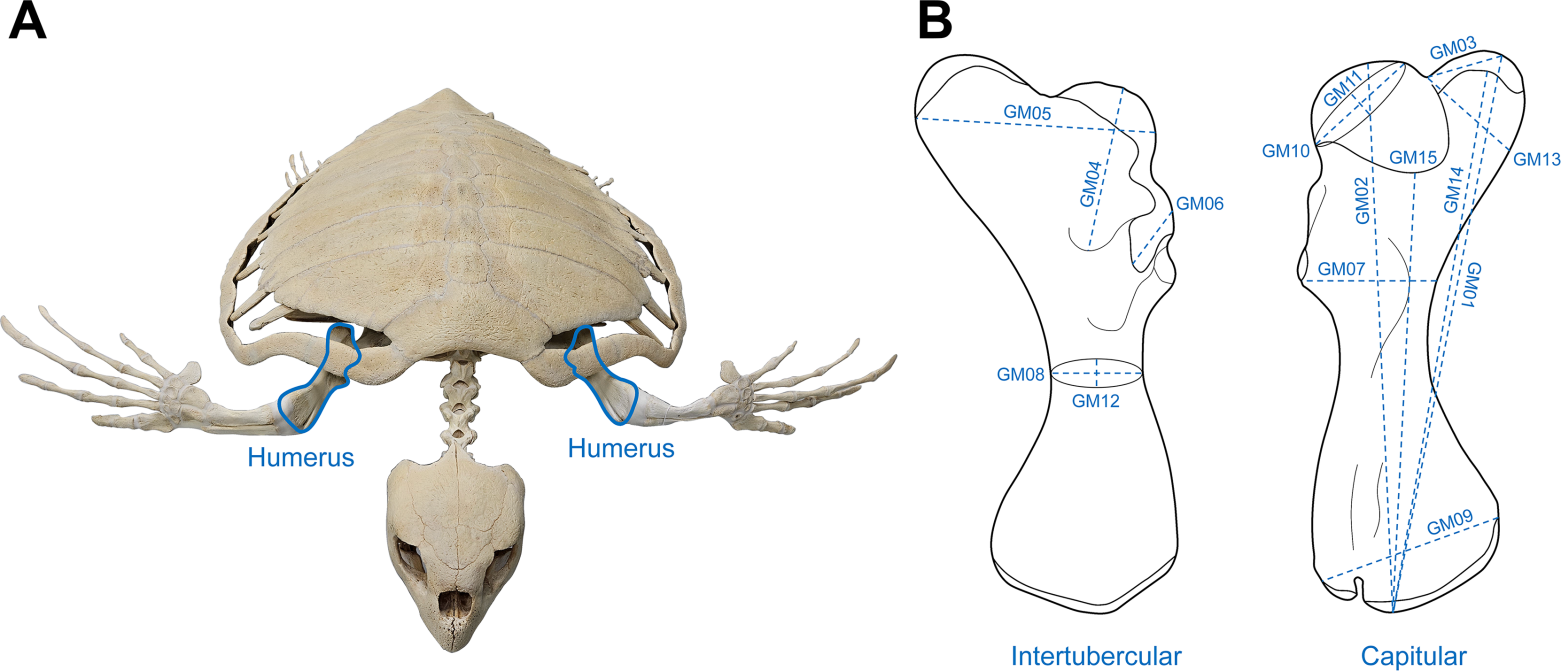
Humerus of sea turtles and gross morphological (GM) measurements. (A) Position of the humerus in a green turtle skeleton and (B) schematic diagram of the intertubercular and capitulard views of the left humerus of the loggerhead turtle. GM01, maximal length; GM02, length along the shaft; GM03, medial process length; GM04, proximal length; GM05, proximal width; GM06, lateral process length; GM07, width at deltopectoral crest; GM08, medial width; GM09, distal width; GM10, maximal head diameter; GM11, minimal head diameter; GM12, shaft thickness; GM13, ulnar process width; GM14, maximal bone length; and GM15, longitudinal bone length. A detailed description of the measurement is provided in Table S1.

After measuring the external features of the sea turtles, we transferred the samples to the autopsy room of the National Institute of Ecology, Seocheon, Republic of Korea. We dissected the carcasses and excised the left humerus. Sea turtle collection and autopsies were approved by the Marine Animal Protection Committee of the Ministry of Oceans and Fisheries (approval number MAB-23-02R). The humeri were pretreated according to the protocol described by Goshe et al. (2020). We separated the adhered flesh and cartilage from the humerus using a knife. The remaining tissues were removed after tenderizing them in water at 60 °C for approximately 6 h in a water bath (Memmert, WNB 22, Schwabach, Germany). After completing the separation, the humeri were dried in the sun for approximately 2 weeks. We measured 15 gross morphological (GM) traits of the humeri (Fig. 2), as described in Abell et al. (2023), Hermanson et al. (2024), and Zug & Parham (1996), using digital calipers (ISO-9000, Mitutyoyo Corp., Tokyo, Japan). The humeri were also weighed using a digital scale (SW-1S, CAS, Yangju, Republic of Korea).

We provided the detailed photographs of samples in the publicly accessible data repository, MorphoSource (https://www.morphosource.org/). To ensure clear detail under high magnification, we acquired a mean of eight images with different focal points for focus stacking using a Nikon D5 camera (Nikon Corporation, Tokyo, Japan). These images were synthesized into a single final image, providing a clear visualization of the entire specimen, utilizing the Helicon Remote software (Helicon Soft Limited, Kharkiv, Ukraine).

### Correlation between the humerus and carapace length

To estimate the SCL, the most representative physical measurement of sea turtles based on the humerus, correlations between the SCL and two humeral measurements—GM01 for length and GM08 for width—were assessed. We calculated the Pearson correlation coefficient of the SCL and the two GMs after conducting the Shapiro–Wilk test to confirm normality. We then analyzed the linear regression patterns between the SCL and the two GMs.

### Interspecific comparison

To compare the differences in carapace, plastron, and humerus shape between loggerhead and green turtles, we standardized measurements based on SCL. First, we standardized three physical sites (SCW, CPL, and CPW) based on SCL; SCW/SCL, CPL/SCL, and CPW/SCL. Second, we standardized 15 other humerus measurement sites (from GM01 to GM15) based on SCL. After conducting the Shapiro–Wilk test, we performed a t-test to compare interspecific differences in the 18 standardized variables. Considering the small sample size and the linear relationship between the humerus and SCL, we did not consider intraspecific differences. We also excluded the weight of the humerus from the comparison because it was highly variable.

Additionally, to determine the primary distinct factors in the shape of the humerus between the two species, we conducted the principal component analysis (PCA) using the 15 SCL-standardized GMs. The data were assessed for suitability in a PCA using the Kaiser-Meyer-Olkin (KMO) and Bartlett’s tests. Since the KMO value was 0.735 (moderate), and the value of the Bartlett’s test was significant (chi-square = 681.919, *P* < 0.001), we assessed this data set as proper for conducting PCA (Shrestha, 2021). We reported only the top principal components with eigenvalues of ≥1. The *prcomp* package in R (R Core Team, 2016) was used for statistical analysis.

## Results

### Carapace, plastron, and humerus measurements

We collected 22 female and six male loggerhead turtles, of which the mean (± standard deviation, SD) measurements were 75.3 ± 6.8 cm at SCL, 62.6 ± 5.4 cm at SCW, 61.2 ± 5.0 cm at CPL, and 46.3 ± 69.0 cm at CPW (Table 1). We collected five female, one male, and eight juvenile green turtles, whose mean measurements were 58.2 ± 15.2 cm at SCL, 48.3 ± 10.4 cm at SCW, 51.0 ± 13.2 cm at CPL, and 44.8 ± 10.2 cm at CPW. Loggerhead turtles had a mean of 161.5 ± 16.5 mm for GM01 and 28.7 ± 2.9 mm for GM08. Green turtles had a mean of 120.9 ± 33.0 mm for GM01 and 25.3 ± 6.3 mm for GM08. The remaining humeral measurements are shown in Table 2, and individual measurements are presented in the Raw Data.

**Table 1:** Mean values of physical measurements of sampled sea turtles with standard deviation and range. All units are centimeters. SCL, straight carapace length; SCW, straight carapace width; CPL, curved plastron length; CPW, curved plastron width.

Species	Sex/Growth	SCL	SCW	CPL	CPW
Loggerhead turtle	Female (*n* = 22)	73.2 ± 5.3	62.1 ± 5.5	60.6 ± 4.8	58.7 ± 4.0
		(62.0–84.4)	(53.0–78.9)	(53.0–72.2)	(46.3–69.0)
	Male (*n* = 6)	82.5 ± 5.6	64.7 ± 4.5	63.2 ± 4.9	62.2 ± 5.4
		(74.3–89.1)	(56.8–68.4)	(54.4–67.5)	(52.3–66.7)
	Total (*n* = 28)	75.3 ± 6.8	62.6 ± 5.4	61.2 ± 5.0	59.5 ± 5.1
		(62.0–89.1)	(53.0–78.9)	(53.0–72.2)	(46.3–69.0)
Green turtle	Female (*n* = 5)	75.7 ± 4.5	60.0 ± 3.6	65.4 ± 3.0	58.3 ± 6.3
		(69.9–79.6)	(54.7–62.1)	(61.2–68.0)	(50.8–64.1)
	Male (*n* = 1)	72.1	56.0	64.9	54.8
	Juvenile (*n* = 8)	48.0 ± 6.0	41.5 ± 6.5	42.1 ± 7.7	38.4 ± 5.9
		(41.3–60.4)	(35.3–56.1)	(34.0–59.7)	(32.0–52.1)
	Total (*n* = 14)	58.4 ± 14.6	48.3 ± 10.4	51.0 ± 13.2	45.8 ± 11.2
		(41.3–79.6)	(35.3–62.1)	(34.0–68.0)	(32.0–64.1)

**Table 2:** Mean length (± standard deviation; in mm) of 15 measurement sites and the mean weight (± SD; in g) of the humerus.

Species	GM01	GM02	GM03	GM04	GM05	GM06	GM07	GM08
Loggerhead	161.5 ± 16.5	152.5 ± 14.5	24.9 ± 3.4	58.2 ± 63.3	63.3 ± 7.3	24.9 ± 2.8	40.6 ± 4.2	28.7 ± 2.9
Green	120.9 ± 33.0	113.9 ± 31.4	20.8 ± 5.8	48.2 ± 13.5	53.0 ± 13.9	21.5 ± 6.2	34.9 ± 9.2	25.3 ± 6.3
*	*t* = 4.176, *P* < 0.001	*t* = 4.479, *P* < 0.001	*P* > 0.05	*P* > 0.05	*t* = −2.190, *P* < 0.05	*t* = −2.754, *P* < 0.01	*t* = −3.265, *P* < 0.01	*t* = −5.393, *P* < 0.001
**Species**	**GM09**	**GM10**	**GM11**	**GM12**	**GM13**	**GM14**	**GM15**	**Weight**
Loggerhead	56.6 ± 6.9	40.7 ± 4.8	31.1 ± 3.8	14.0 ± 1.5	33.1 ± 3.5	144.9 ± 14.7	117.4 ± 10.9	99.8 ± 36.5
Green	42.0 ± 9.8	34.1 ± 8.9	27.0 ± 7.6	12.4 ± 2.9	27.7 ± 6.7	107.2 ± 28.7	88.1 ± 21.9	70.7 ± 46.9
*	*t* = 3.095, *P* < 0.01	*t* = −2.109, *P* < 0.05	*t* = −2.934, *P* < 0.01	*t* = −5.243, *P* < 0.001	*t* = −2.294, *P* < 0.05	*t* = 4.761, *P* < 0.001	*t* = 3.439, *P* < 0.01	–

**Note:**

* Results from the t-test comparing the SCL-standardized measurements; df, 38 in all comparisons.

### Correlation between the humerus and carapace

**Figure 3:**
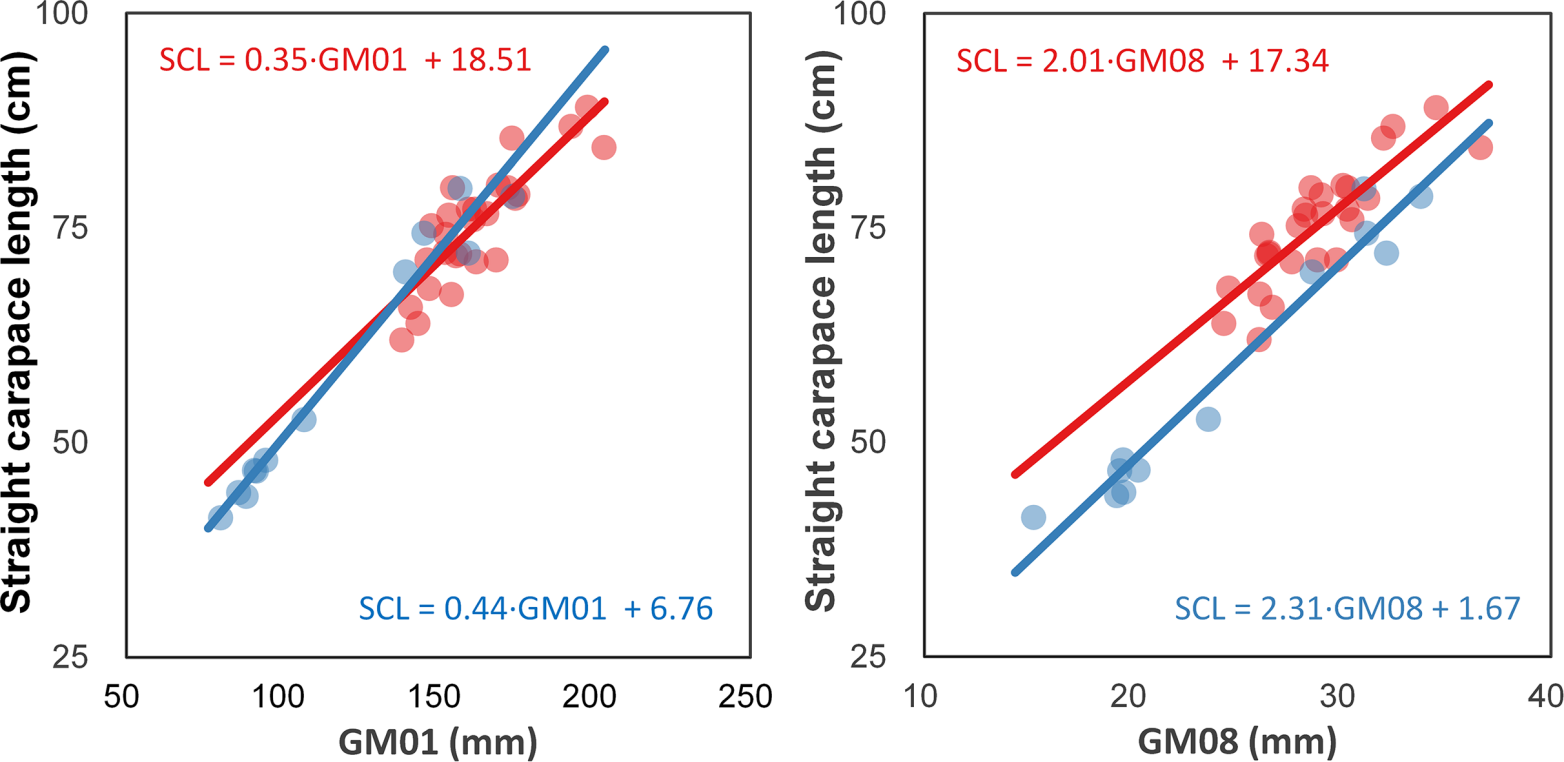
Correlation between straight carapace length (SCL) and the length and width of the humerus from loggerhead and green turtles. GM01 and GM08 represent the length and width of the humerus, respectively.

We found significant correlations between the SCL and two GMs for the two sea turtle species (Fig. 3). The SCL of loggerhead turtles was positively correlated with GM01 (*r*^*2*^ = 0.710, *P* < 0.001) and GM08 (*r*^*2*^ = 0.713, *P* < 0.001). The SCL of green turtles was also positively correlated with GM01 (*r*^*2*^ = 0.966, *P* < 0.001) and GM08 (*r*^*2*^ = 0.954, *P* < 0.001).

The regression equation for SCL between GM01 was SCL = 0.35 × GM01 + 18.51 at loggerhead turtles and SCL = 0.44 × GM01 + 6.76 at green turtles. The regression equation for SCL between GM08 was 2.01 × GM08 + 17.34 at loggerhead turtles and SCL = 2.31 × GM08 + 11.67 at green turtles.

### Interspecific differences in measurements

**Figure 4:**
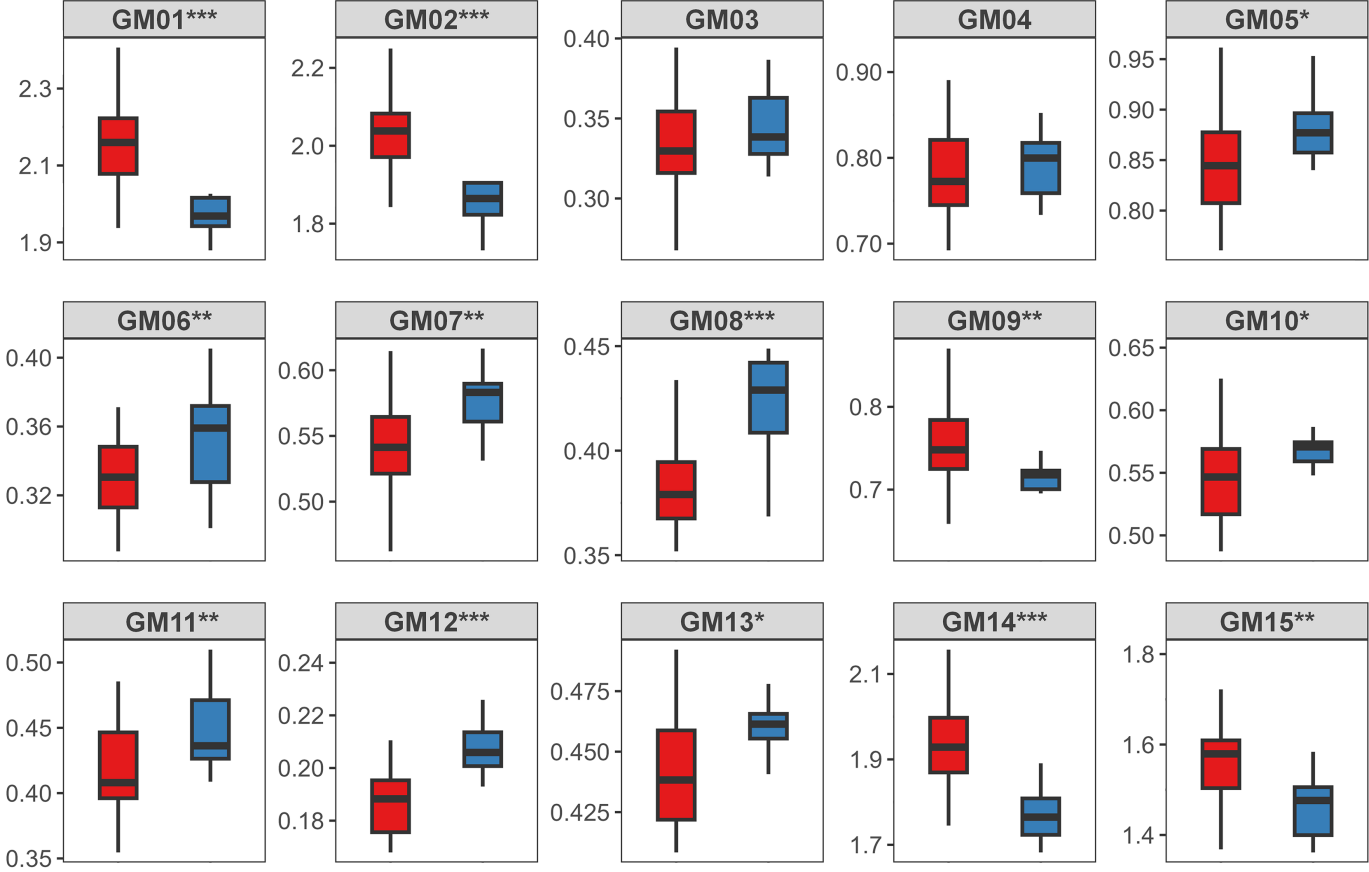
The interspecific comparison of straight carapace length-standardized gross morphological (GM) measurements of the humerus between loggerhead (red boxes) and green (blue boxes) turtles. The asterisks indicate the degree of significance (*P < 0.05; **P < 0.01, ***P < 0.001). See Fig. 1’s caption for a description of each measurement.

The green turtles had a longer CPL than the loggerhead turtles (*t* = −3.109, *P* < 0.01), whereas the SCW and CPW did not differ significantly between the two species (*P*s > 0.05). A comparison of the SCL-standardized humerus measurements revealed that only GM03 and GM04 did not differ significantly (*P*s > 0.05), while all other GMs differed significantly between the two species (Fig. 4).

**Figure 5:**
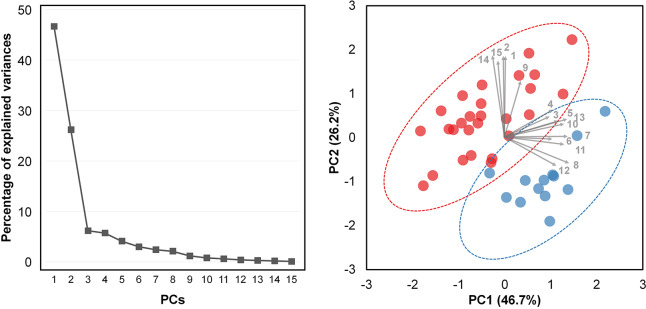
Principal component score plot of the straight carapace length-standardized gross morphological measurements of the humerus of loggerhead (red dot) and green (blue dot) turtles. Each arrow number indicates the gross morphological (GM) measurement number.

Two principal component (PCs) with a cumulative explanatory value of 72.9% were extracted from the PCA results (Table 3, Fig. 5). The two major principal components, PC1 and PC2, explained 46.7% and 26.2% of the variance, respectively. The dominant factors in PC1 were GM04, GM05, and GM10, which correlated with humeral width, while the major factors in PC2 were GM01, GM02, GM14, and GM15, which were associated with humeral length (Fig. 5).

**Table 3:** Information on principal component analysis of the straight carapace length-standardized gross humeral measurements of the left humerus. The two top principal components have eigenvalues above one.

Component	PC1	PC2
GM01	0.592	0.771
GM02	0.576	0.778
GM03	0.718	−0.060
GM04	0.818	−0.013
GM05	0.929	−0.182
GM06	0.595	−0.295
GM07	0.803	−0.354
GM08	0.635	−0.624
GM09	0.623	0.449
GM10	0.853	−0.212
GM11	0.706	−0.413
GM12	0.448	−0.574
GM13	0.797	−0.149
GM14	0.439	0.853
GM15	0.476	0.773
Eigenvalue	7.00	3.93
Variation explained (%)	46.7	26.2

**Note:**

GM, gross measurement; PC, principal component.

## Discussion

The length and width of the humeri of loggerhead and green turtles were positively linearly correlated with SCL. This trend is consistent with previous studies, which have shown that the increase in humeral diameter of the two sea turtle species closely matches the SCL growth rate (Goshe et al., 2010; Petitet et al., 2012), suggesting that estimating SCL based on the humerus is a reasonable approach. The SCL estimation based on the humerus can complement information on the carapace of damaged or decomposed sea turtle carcasses. Korean waters are located at the northern range limit of loggerhead and green turtles, whose populations are significantly smaller than those in the southern tropical and subtropical regions, the main habitats of sea turtles. In addition to the small sample size, some individuals are often found to have even damaged or severely decayed carapaces due to ship collisions, limiting size measurements (Kim et al., 2020; Park, Park & Kim, 2025). In Korean waters, individual information and population sizes are collected primarily from stranded sea turtle carcasses or those caught as bycatch, rather than from living turtles (Kim et al., 2024; Park, Park & Kim, 2025). Thus, in regions such as Korean waters, where sampling sea turtles is challenging, humerus-based size estimation methods could help reduce the gaps in sea turtle size data. However, our results only provide information for sea turtles with high annual growth rates. Adult loggerhead and green turtles are known to grow to approximately 105 and 100 cm of SCL, respectively (Hayashi & Nishizawa, 2015). In addition, both loggerhead (Casale et al., 2011; Casale, Mazaris & Freggi, 2011) and green (Goshe et al., 2010; Eguchi et al., 2012) turtles exhibit a decline in annual growth rate once they reach an SCL of approximately 60–80 cm. Because the loggerhead and green turtles collected in this study were individuals with a relatively high growth rate (mean SCL of 62.6 cm and 58.2 cm, respectively), the analysis of turtles with a reduced annual growth rate was limited. Considering that loggerhead and green turtles using Korean water belong to the Northwest Pacific management units (Park et al., 2025) and the Japan management unit (Park et al., 2024), respectively, including samples from Japan and Taiwan, where the two adult sea turtle species primarily occur, could enhance SCL estimation based on the humerus.

At equivalent SCLs, green turtles had a thicker humerus at the shoulder, whereas loggerhead turtles had a longer overall length and a thicker humerus toward the distal end. GM04, GM05, and GM10, which had high scores in PC1, were all related to the thickness of the area connected to the shoulder and were similar or greater in the green turtles than the loggerheads, whereas GM09, the distal thickness of the humerus, was greater in loggerhead turtles. The most important measurements in PC2 were primarily related to the total length of the humerus, and loggerhead turtles had higher values for these measurements. Given that loggerhead and green turtles sympatrically use Korean waters (Park, Park & Kim, 2025) and both originate from Japanese rookeries (Park et al., 2024, 2025), interspecific differences in humeral shape may be related to their distinct migration patterns rather than their habitats. The flippers of sea turtles are biomechanically designed to swim efficiently by passive upstroke through the current, as well as by flapping to generate propulsion (van der Geest et al., 2022). Loggerhead turtles, which migrate much longer distances than green turtles, may benefit from using ocean currents more effectively than by active swimming. Most loggerhead turtles in Korean waters originate from Japanese nesting grounds (Park et al., 2025). Loggerhead turtles originating from Japanese rookeries migrate to the Pacific Ocean *via* the Kuroshio and North Pacific currents immediately after hatching, travel over 10,000 km, and spend time in the eastern Pacific along the coasts of the United States and Mexico until they reach juvenile or subadult status (Bowen et al., 1995; Abecassis et al., 2013). After maturing, loggerhead turtles return to the western Pacific Ocean (Abecassis et al., 2013). Because it is inefficient to migrate such extremely long distances by only active flapping, loggerhead turtles may have developed long and broad-tipped fore flippers, like oars, to use ocean currents. Similarly, the leatherback turtle (*Dermochelys coriacea*), the largest and most mobile of all sea turtles, has the longest and thinnest humerus among sea turtles (Nakajima, Hirayama & Endo, 2014). While the correlation between elongated humeri and high utilization of ocean currents in both sea turtle species was not definitively verified, further analysis could improve our understanding on the connection between osteologic and ecological traits.

In contrast, green turtles had shorter and thicker humeri near the shoulder than loggerhead turtles, possibly because they are active swimmers that engage in more flapping. Green turtles swim more vigorously and faster than loggerhead turtles (Smith & Salmon, 2009; Pereira, Booth & Limpus, 2011; Shiode et al., 2021). In water, large bodies experience greater flow resistance than small ones and uptake due to active swimming places high levels of stress on the front flippers of large sea turtles (Vogel, 2008; Nishizawa, Asahara & Kamezaki, 2013). Green turtles are known to have a smaller carapace than loggerheads (Hayashi & Nishizawa, 2015), and their relatively short and thick humeri can withstand the stress of flapping. However, they may be less efficient than loggerhead turtles at using ocean currents. Although green turtles use ocean currents for migration (Cheng & Wang, 2009), the utilization rate appears to be lower than that of loggerhead turtles, which use large ocean currents (Abecassis et al., 2013). In the Northwest Pacific, green turtles commonly migrate shorter distances than loggerhead turtles and move north-south between habitats, as confirmed by satellite tracking (Kim et al., 2022, 2024) and inter-population gene sharing (Hamabata, Kamezaki & Hikida, 2014; Park et al., 2024). In this region, green turtles primarily travel along shallow coastal waters (Kim et al., 2022, 2024), and sometimes swim against ocean currents (Luschi, Hays & Papi, 2003). Therefore, green turtles may have shorter and thicker humeri, which enable vigorous flapping and a relatively low utilization rate of ocean currents compared with loggerhead turtles.

Although the evolutionary history of sea turtle flippers is highly related to their limb mobility (Fujii et al., 2018), the differences in their humeri between loggerhead and green turtles may be related to their habitats or other characteristics, in addition to their migration habits. Consistent with previous studies (Jang et al., 2018; Kim et al., 2024; Park, Park & Kim, 2025), loggerhead turtles were collected mainly from the eastern Korean Peninsula, whereas green turtles were mainly sampled from Jeju Island. Although the eastern sea of the Korean Peninsula has a lower sea temperature than the Jeju Sea, making it less suitable for body temperature regulation, the formation of an oceanic front provides an abundant food source for sea turtles (Kim et al., 2024; Park, Park & Kim, 2025). The humerus of loggerhead turtles may have evolved to increase energy efficiency for locomotion in colder feeding grounds. Additionally, the stress on the humerus during terrestrial movements should be considered. Although sea turtles spend most of their lifetime in the ocean, gravid females occasionally come ashore to nest (Carr & Ogren, 1959). On land, sea turtles rely almost entirely on their forelimbs for locomotion, and body mass is a key factor in the stress placed on the humerus during movement (Dickson & Pierce, 2019; Nishizawa, Asahara & Kamezaki, 2013). However, our samples were collected from strandings or bycatch, limiting analysis using body mass. In addition, collecting information on sea turtle locomotion on the Korean coast is challenging, as it is not a primary nesting ground, despite sporadic reports of nesting (Jung et al., 2012). Considering the lack of understanding of the locomotion mechanism of sea turtles on land, analyzing related data, such as humeral thickness and density, body mass, and terrestrial movement distance, will provide new insights into the structure of the humerus.

## Conclusions

This study presents morphological information on the humeri of sympatric loggerhead and green turtles occurring in Korean waters, as well as their interspecific differences. We propose that the differences in the length and thickness of the humerus between the two sea turtle species may be attributable to their migration patterns and usage rates in oceanic currents. This also means that the structure and function of body parts may vary depending on the species’ use of its habitat and response to its environment, which could be identified through osteological analysis. Although we analyzed the standardized humerus values and presented a linear correlation between the SCL and the humerus to ensure comparative validity for interspecific comparison, it should be noted that the results were based on biased samples. Collecting sufficient samples for sexual comparison of Korean sea turtles was limited, because mainly female loggerhead and green turtles are observed in Korean waters (Park et al., 2024, 2025). These limitations must be addressed through consistent data collection based on the criteria presented in this study. Furthermore, our findings confirmed that estimating sea turtle SCL from humeral data can help compensate for missing individual measurements in areas with challenging sampling conditions at distributional boundaries. Sea turtle range shifts have been observed in many areas following recent climate change, particularly at the northern range limit of sea turtles (Mancino, Canestrelli & Maiorano, 2022; Luna-Ortiz et al., 2024), including Korean waters (Park, Park & Kim, 2025). The accumulation of robust data is expected to aid the quantitative analysis of sea turtle populations and ecology in this region, providing a cornerstone for the development of conservation and management strategies.

## Supplemental Information

10.7717/peerj.20958/supp-1 Supplemental Information 1 Raw data.

10.7717/peerj.20958/supp-2 Supplemental Information 2 Explanation of humeral bone gross morphological (GM) measurements. This criterion followed (Abell et al., 2023).

10.7717/peerj.20958/supp-3 Supplemental Information 3 MorphoSource Specimen DOIs.

## Additional Information and Declarations

### Data Availability

The following information was supplied regarding data availability:

The raw data is available in the Supplemental Files.

Detailed photographs of humeral samples are available at MorphoSource:

https://www.morphosource.org/projects/000802646?locale=en.

- The complete list of MorphoSource specimen DOIs is available in the Supplemental Files.
