# Supplementary material for: Osteological differences in the humerus of loggerhead and green turtles
Source: PeerJ. 2026 Apr 20;14:e20958. doi: 10.7717/peerj.20958 (PMC13105183; doi:10.7717/peerj.20958)
Supplement: Supplemental Information 2 — This criterion followed (Abell et al., 2023). [file peerj-14-20958-s002.docx]

**Supplemental Table 1.** Explanation of humeral bone gross morphological (GM) measurements. This criterion followed Abell et al. (2023) and Hermanson et al. (2024).

| **Measurement** | **Name** | **Description** |
| --- | --- | --- |
| GM01 | Maximal length | Distance from the proximal tip of the ulnar process to the distal articular cartilage surface |
| GM02 | Length along the shaft | Distance from proximal cartilage surface of head to distal articular surface of cartilage (parallel to longitudinal axis) |
| GM03 | Medial process length | Distance from the proximal tip of the ulnar process to the juncture of the head and process |
| GM04 | Proximal length | Distance from proximal surface of head to distal edge of secondary deltopectoral crest (parallel to longitudinal axis) |
| GM05 | Proximal width | Distance from preaxial surface of head to postaxial surface of ulnar process (perpendicular to longitudinal axis) |
| GM06 | Lateral process length | Distance from pre- to postaxial edges of radial process, diagonal to longitudinal axis |
| GM07 | Width at deltopectoral crest | Transverse distance of shaft from pre- to postaxial surfaces at deltopectoral crest |
| GM08 | Medial width | Transverse distance from pre- to postaxial surfaces at the point of minimum width |
| GM09 | Distal width | Transverse distance from pre- to postaxial surfaces at the juncture of articular condyles with the shaft |
| GM10 | Maximal head diameter | Maximal diameter of the head |
| GM11 | Minimal head diameter | Minimal diameter of the head |
| GM12 | Shaft Thickness | Minimal depth in the middle of the shaft, in the vicinity of the medial width, perpendicular to the longitudinal axis |
| GM13 | Ulnar process width | Distance from postaxial surface of ulnar process to juncture of head and process, diagonal to longitudinal axis |
| GM14 | Maximal bone length | Distance from the bone at the proximal tip of the ulnar process to the distal articular bone surface |
| GM15 | Longitudinal bone length | Distance from the proximal surface of the bone at the head to the distal articular surface of the bone |
